# Using Mobile Devices for Vocabulary Learning Outside the Classroom: Improving the English as Foreign Language Learners’ Knowledge of High-Frequency Words

**DOI:** 10.3389/fpsyg.2022.899885

**Published:** 2022-06-24

**Authors:** Azadeh Rahmani, Vahid Asadi, Ismail Xodabande

**Affiliations:** ^1^Department of Foreign Languages, Danesh Alborz University, Takestan, Iran; ^2^Department of Foreign Languages, Islamic Azad University, Yasuj, Yasuj, Iran; ^3^Department of Foreign Languages, Kharazmi University, Tehran, Iran

**Keywords:** mobile assisted language learning, digital flashcards, vocabulary – general vocabulary, English as a foreign language, word list learning

## Abstract

The present study investigated the impacts of mobile assisted vocabulary learning *via* digital flashcards (DFs). The data were collected from 44 adult English as Foreign Language (EFL) learners in three intact classes in a private language teaching institute in Iran, randomly assigned to experimental (*N* = 27) and control (*N* = 17) learning conditions. The experimental group used a freely available DF application (i.e., NGSL builder) to learn items from a recently developed corpus-based word list for high-frequency vocabulary in English (NGSL). The treatment was implemented as out-of-the-classroom learning activities where the EFL learners used DFs to augment their vocabulary knowledge, and their learning gains were compared to the control group that received regular English language education. The participants’ vocabulary knowledge was tested in pre-, post-, and delayed post-tests, and the findings indicated that using DFs for outside the classroom vocabulary learning contributed significantly to short- and long-term improvements in the knowledge of high-frequency words. The study provided empirical evidence for the affordances of mobile assisted vocabulary learning for learning a considerable proportion of core vocabulary and has some implications for addressing the vocabulary learning needs of EFL learners.

## Introduction

The importance of vocabulary in second language literacy developments is widely acknowledged (Nation, 2013; Webb and Nation, 2017). More specifically, vocabulary knowledge is considered crucial for language learners’ proficiency development in the four macro skills including reading, listening, speaking, and writing (Clenton and Booth(eds), 2020), and regarded to be as the single most important variable in language learning in general (Dodigovic and Agustín-Llach, 2020). In light of these considerations, a major pedagogical challenge in teaching English to speakers of other languages (TESOL) has been addressing the vocabulary learning needs of learners at different stages of language learning (Nation, 2013). Over the past decades, this consistent concern resulted in the creation of a number of corpus-based word lists for high-frequency and academic vocabulary to inform language teaching materials developments (Coxhead, 2000; Gardner and Davies, 2014; Brezina and Gablasova, 2015; Browne, 2021; Cobb and Laufer, 2021). High-frequency or general service words are commonly used vocabulary items in spoken and written language that provide from 80 to 90% coverage in most texts in English. The academic vocabulary, however, refers to a group of words for describing abstract ideas and processes in academic discourse (Coxhead, 2000). Given the large coverage and hence the importance of high-frequency words, there is a need for systematically focusing on teaching them and finding effective strategies to facilitate their acquisition by language learners. Nonetheless, vocabulary instruction generally remained underestimated in second language classrooms (Dodigovic and Agustín-Llach, 2020), and language teachers mostly pay insufficient attention to it for some reasons such as shortage of classroom time and high workloads (Webb and Nation, 2017).

With significant developments in Information and Communication Technologies, there is a growing interest in TESOL to use various affordances provided by digital technologies for vocabulary learning (Burston and Giannakou, 2021; Hao et al., 2021; Yang et al., 2021; Yu and Trainin, 2021). The expanding body of knowledge in this line of inquiry indicated that technology assisted learning is generally facilitative for second language vocabulary development (Mahdi, 2017; Lin and Lin, 2019). Accordingly, the integration of mobile assisted vocabulary learning in TESOL provide opportunities for a systematic focus on teaching the vocabulary items that are essential for the communication needs of language learners (such as high-frequency words; Xodabande et al., 2022). Additionally, the implementation of technological resources to augment vocabulary development might be more effective for outside the classroom learning conditions, as in most cases formal educational settings are resistant to integrating new technologies into the established curricula and teaching methodologies (Lai and Gu, 2011). Furthermore, a unique feature of digital technologies with considerable potential for transforming education is the possibility of extending learning and teaching to anytime and anyplace. In this regard, complementing classroom instruction with beyond the classroom technology assisted activities gives more freedom to students to conduct self-directed learning and also helps them to become more autonomous learners which is a valuable educational asset (Xodabande et al., 2022).

Among the various strategies developed for learning words, flashcards provide learners with a fast and effective way to improve their vocabulary knowledge (Nation, 2013). With the growing availability of smartphone devices for language learners, digital flashcards (DFs) attracted increased interest from researchers in recent years (Sage et al., 2019; Xodabande and Atai, 2020; Yüksel et al., 2020; Xodabande et al., 2022). Collectively, research in this area indicated that DFs used on mobile or computer devices contributed significantly to language learners’ vocabulary development and increased their motivation and engagement with the learning materials. DFs also proved to be more effective than paper- or computer-based cards mainly because of their accessibility and portability as learning tools. Researchers also investigated the effectiveness of DFs for learning academic and technical vocabulary, and also as an instrument for autonomous and self-directed vocabulary learning. Despite significant positive learning outcomes, most of the studies in this line of research were conducted in short time periods with interventions targeting a limited number of vocabulary items (Xodabande and Atai, 2020). These limitations make it difficult to understand the long-term impacts of mobile assisted vocabulary learning in general (Lin and Lin, 2019), and the potential of such emerging learning environments for learning a large number of words in particular (Xodabande et al., 2022). Accordingly, there is a need for more empirical research to explore the affordances of mobile devices and DFs for learning vocabulary items in the corpus-based word lists, and to track the learning outcomes in the long term through delayed post-tests.

The present study aimed to facilitate the learning of high-frequency vocabulary by English as Foreign Language (EFL) learners *via* mobile devices and DFs. To this end, the high-frequency vocabulary in English is operationalized based on New General Service List (NGSL) project (Browne, 2021) which is among the most recently developed corpus-based core vocabulary lists. The NGSL contains 2,818 items (i.e., flemmas) derived from a 273 million-word subsection of Cambridge English Corpus. The pedagogical value of the NGSL arises from its considerable coverage (around 92.34%) of the corpus used for developing the list. Accordingly, mastery over the NGSL is essential for achieving the minimum threshold for text comprehension in English (Laufer, 2013), which might be regarded as the first step in developing the lexical knowledge of EFL learners. Furthermore, given the existing research-practice gap in corpus-based language education (Chambers, 2019), the current study also aimed to connect research in wordlist development to the language learning needs of EFL learners and investigated both short- and long-term impacts of mobile assisted vocabulary learning. This is particularly noteworthy as in most EFL learning contexts, many language learners fail to acquire the high-frequency vocabulary after years of language education (Webb and Chang, 2012), and interventions that have lasting impacts on their vocabulary knowledge contribute significantly to their second language literacy development. The following research questions were addressed in this study:

1. Does using DFs for outside the classroom mobile assisted vocabulary learning result in boosting EFL learners’ vocabulary knowledge?
2. Does mobile assisted vocabulary learning have long-term impacts on vocabulary learning as assessed by a delayed post-test?

## Method

### Participants

Data for the present study were collected from three intact classes in a private language teaching institute in Iran. The participants were 44 adult language learners (16 males, 25 females) selected based on their availability in the research context, and their ages ranged from 17 to 34 (*M* = 23). According to the results of the Cambridge online placement test (Test Your English, 2022), most of the participants were in B1 level (pre-intermediate) proficiency in English as outlined in the Common European Framework of Reference (Council of Europe, 2001). Two classes were randomly assigned to the experimental conditions (*N* = 27) and one class acted as a control group (*N* = 17). In compliance with ethical considerations in educational research, informed consents were obtained from the participants and they were informed regarding the goals of the research, stages of data collection, and confidentiality of personal information collected in the study.

### Materials and Testing Instruments

The study used a freely available DFs application (i.e., NGSL builder; EFL Technologies, 2017) which is designed for learning high-frequency vocabulary items in the NGSL (Browne, 2021). The NGSL builder application allows learners to select vocabulary items in different levels from beginner (0% skipped) to advanced (75% skipped). The application also supports using DFs in receptive (meaning recall) and productive (form recall) learning modes. Additionally, the NGSL provides part of speech information and North American pronunciation for the words and has a built-in feature for recycling learned items in specific time intervals (i.e., spaced repetition). After an initial assessment of the vocabulary knowledge in the pre-test, 50% of NGSL words were skipped for the participants. For testing the vocabulary knowledge of the participants before and after the treatment, the study used NGSL Test (Stoeckel and Bennett, n.d.) which is a standard and validated measure of the NGSL with high reliability (Cronbach’s alpha = 0.90). The test contains 100 items grouped in five levels and each level contains 20 multiple response questions.

### Procedures and Data Analysis

Prior to starting the treatment, the participants’ vocabulary knowledge was tested using the NGSLT. Following this initial vocabulary learning needs identification, those participants in the experimental learning condition (classes A and B) installed the NGSL Builder application on their smartphone devices. The participants in these two classes received 2 h of training (in two online sessions) on using DFs for outside the classroom vocabulary learning and learned how to email their progress to the researchers through a feature available in the NGSL builder app. The treatment was implemented in a 3-month period where the participants were enrolled in a language learning course (focusing on developing communicative language ability) in a private language teaching institute in the context of the study. Accordingly, the participants in the control group (class C) received their regular education, while the participants in the experimental group used the NGSL builder application outside the classroom as a tool for boosting their vocabulary knowledge. The three classes had one and the same teacher, were taught based on a pre-specified syllabus determined by the language teaching institute, and identical materials and methods were used to teach the classes. The vocabulary knowledge of the participants was tested again at the end of the training period, and a follow-up delayed post-test was administered 3 months later. The scores on vocabulary tests were analyzed using IBM SPSS (version 25) for descriptive and inferential statistics. In this regard, after running descriptive statistics to identify mean scores for different learning conditions, a mixed between-within subjects analysis of variance (ANOVA; Pallant, 2016) was performed to further investigate the main effects for each of the independent variables (i.e., group with two levels and time with three levels).

## Results

**FIGURE 1:**
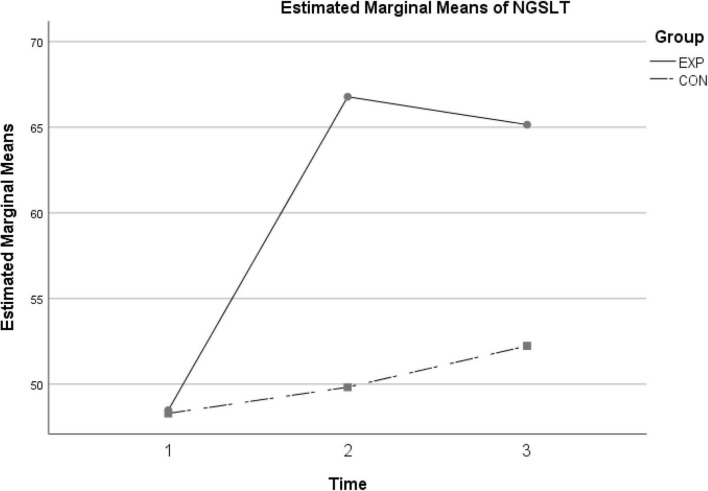
Mean scores for the NGSLT across time.

The results of the descriptive statistics for the obtained scores by the two groups indicated that for test 1 (i.e., pre-test), the participants in the experimental and control group had similar scores on NGSLT, and the total mean value was calculated as 48.41 (*SD* = 5.16). In order to check for any possible differences in vocabulary knowledge prior to treatment, an independent samples *t*-test was conducted that yielded non-significant results [*t* (42) = 0.116, *p* = 0.908, two tailed]. Nevertheless, in test 2 (i.e., post-test), the participants in the experimental group obtained higher scores (*M* = 66.78, *SD* = 4.31) compared to the control group (*M* = 49.82, *SD* = 5.97). Similar results were obtained in test 3 (i.e., delayed post-test) and the participants in the experimental group (*M* = 65.15, *SD* = 4.71) scored higher than the control group (*M* = 52.24, *SD* = 6.33). In order to further analyze the scores for statistical differences, a mixed between-within subjects ANOVA was conducted. Prior to investigating the main effects, preliminary analyses including Levene’s test of equality of error variances (assumption of homogeneity of variances) and Box’s test of equality of covariance matrices (assumption equality of covariance matrices) were examined and no violation of the assumptions required for ANOVA was observed. The results obtained for multivariate tests (Table 1) revealed a significant interaction effect between the two variables (i.e., time and group; Wilks’ Lambda = 0.39, *F* (2, 41) = 32.04, *p* = 0.000, η_*p*_^2^ = 0.61). This interaction effect means that the changes in the obtained scores over time were different for the experimental and control groups (see Figure 1 below). Moreover, the findings indicated that there is a statistically significant main effect for time (within subjects variable), Wilks’ Lambda = 0.24, *F* (2, 41) = 64.99, *p* ≤ 0.001, η_*p*_^2^ = 0.76. As a result, the changes in the scores were statistically significant across the three testing times, and the findings revealed a very large effect size for the differences (Cohen, 1988). After investigating the main effect for time, the analysis proceeded to explore the between-subjects effects. As it is shown in Table 2, the main effect resulting from the two learning conditions was statistically significant, *F* (1, 42) = 117.56, *p* ≤ 0.001, η_*p*_^2^ = 0.74. Accordingly, the findings indicated that mobile assisted vocabulary learning resulted in significant improvement in the vocabulary knowledge of the participants in the experimental group and the effect size of the difference was very large.

**TABLE 1:** Multivariate tests*^a^*.

Effect		Value	*F*	Hypothesis df	Error df	Sig.	Partial Eta Squared
Time	Pillai’s Trace	0.760	64.991*^b^*	2.000	41.000	0.000	0.760
	Wilks’ Lambda	0.240	64.991*^b^*	2.000	41.000	0.000	0.760
	Hotelling’s Trace	3.170	64.991*^b^*	2.000	41.000	0.000	0.760
	Roy’s Largest Root	3.170	64.991*^b^*	2.000	41.000	0.000	0.760
Time × Group	Pillai’s Trace	0.610	32.044*^b^*	2.000	41.000	0.000	0.610
	Wilks’ Lambda	0.390	32.044*^b^*	2.000	41.000	0.000	0.610
	Hotelling’s Trace	1.563	32.044*^b^*	2.000	41.000	0.000	0.610
	Roy’s Largest Root	1.563	32.044*^b^*	2.000	41.000	0.000	0.610

*^a^Design: Intercept + Group. Within subjects design: Time.*

*^b^Exact statistic.*

**TABLE 2:** Tests of between-subjects effects.

Measure: NGSLT						
Transformed variable: Average						
Source	Type III sum of squares	df	Mean square	*F*	Sig.	Partial Eta Squared
Intercept	380,422.010	1	380,422.010	14,238.700	0.000	0.997
Group	3,140.919	1	3,140.919	117.561	0.000	0.737
Error	1,122.134	42	26.717			

Figure 1 provides visual representation of the scores obtained by the participants over time. Given the significant interaction effect for the pre-test to the post-test, this plot is helpful in interpreting the observed main effects. Accordingly, from the scores obtained by the experimental group is significantly larger. Moreover, from the post-test to the delayed post-test, the scores obtained by the experimental group dropped slightly, nonetheless, the associated mean value was still considerably higher than the mean score obtained by the control group.

In order to investigate the long-term impacts of the intervention, a series of pairwise comparisons were conducted for the scores obtained by the experimental and control groups on NGSLT (Table 3). The results indicated that for the experimental group, the scores on both post- and delayed post-tests were significantly higher than the scores obtained on the pre-test. However, the observed decline in the scores from post-test to the delayed post-test was not statistically significant. As for the control group, the findings indicated that the only significant differences in the scores were between the pre-test and the delayed post-test. These findings provided empirical evidence for the short- and long-term effects of mobile assisted vocabulary learning *via* DFs.

**TABLE 3:** Pairwise comparisons.

Measure: NGSLT							
Group	(I) TIME	(J) TIME	Mean difference (I-J)	Std. error	Sig.*^a^*	95% Confidence interval for difference*^a^*	
						Lower bound	Upper bound
EXP	1	2	−18.296*	1.584	0.000	−22.350	−14.243
		3	−16.667*	1.377	0.000	−20.192	−13.142
	2	1	18.296*	1.584	0.000	14.243	22.350
		3	1.630	1.369	0.734	−1.874	5.134
	3	1	16.667*	1.377	0.000	13.142	20.192
		2	−1.630	1.369	0.734	−5.134	1.874
CON	1	2	−1.529	1.760	1.000	−6.233	3.174
		3	−3.941*	1.048	0.005	−6.743	−1.139
	2	1	1.529	1.760	1.000	−3.174	6.233
		3	−2.412	2.216	0.878	−8.336	3.513
	3	1	3.941*	1.048	0.005	1.139	6.743
		2	2.412	2.216	0.878	−3.513	8.336

*Based on estimated marginal means.*

*The mean difference is significant at the 0.05 level.

*^a^Adjustment for multiple comparisons: Bonferroni.*

## Discussion and Conclusion

The first research question investigated the contribution of mobile assisted vocabulary learning in augmenting the EFL learners’ knowledge of high-frequency vocabulary in English. The findings revealed that outside the classroom vocabulary learning with DFs was effective in boosting the knowledge of core words in English, and the learning outcomes were considerable given the treatment time. More specifically, the participants in the experimental group learned around 18.3% of the NGSL items over the course of 3 months which is equivalent to 500 words. The findings are congruent with previous studies that reported positive learning gains for DFs (Sage et al., 2019; Yüksel et al., 2020; Xodabande et al., 2022). There might be a number of factors contributing to such improvements in vocabulary knowledge. First, these learning gains mostly resulted from the retrieval practice which involves recalling the meaning of the target words from previous encounters (Barcroft, 2007). Such retrievals are associated with developments in both receptive and productive vocabulary knowledge in using paper and DFs (Li and Hafner, 2022). Second, language learners in the experimental group were exposed to a large number of words presented to them in the form of DFs, and given the effectiveness of explicit focus on vocabulary learning (Webb and Nation, 2017), they learned more words compared to the control group whose encounter with words was limited to the materials used in the classes. The use of mobile devices and DFs for vocabulary learning beyond the classroom might have also resulted in increased motivation among the participants in the experimental group, and this inherent motivation associated with new technologies (Stockwell, 2013) impacted learning outcomes.

The second research question was concerned with the long-term effects of mobile assisted vocabulary learning. The results of the delayed post-test indicated that the participants in the experimental group retained most of their acquired knowledge after 3 months following the treatment (albeit with some decline in their vocabulary test scores). This is also in line with the earlier studies that investigated the long-terms impacts of DFs for vocabulary learning (Xodabande et al., 2022). However, the findings of the current study differ slightly from Xodabande and Atai (2020) who found a significant decline in the knowledge of academic vocabulary in the delayed post-test. One reason for this observation might be the nature of target vocabulary items, as the current study focused on high-frequency vocabulary which is more common than academic vocabulary in different text types. This frequency factor enhances the chance of more and varied encounters with target words for EFL learners which is not the case for academic vocabulary which is associated mostly with academic discourse. Additionally, the employed DFs application had a built-in feature for spaced repetition that further contributed to this long-term learning outcomes. Moreover, the EFL learners have more chance to use high-frequency vocabulary than the academic words, and meaningful use of words is essential for long-term improvements in vocabulary knowledge (Webb and Nation, 2017).

The study has some implications for integrating mobile assisted vocabulary learning into TESOL programs. First, as the findings indicated, complementing regular English courses with outside the classroom vocabulary learning with DFs facilitates the bridging of the vocabulary gap commonly reported for EFL learners (Webb and Chang, 2012). Accordingly, instructors might consider using such interventions to help their students in building a good vocabulary base despite limitations in classroom time and language teaching materials. Second, given the considerable learning outcomes (i.e., 18% of the NGSL) for learning with DFs, it seems that mobile assisted vocabulary learning brings opportunities for faster growth in the lexical dimension of second language literacy development which normally takes many years (Webb and Chang, 2012; Nation, 2013; Webb and Nation, 2017). This is specifically important in EFL contexts where learners need to master high-frequency vocabulary as a foundation for language learning in general (Clenton and Booth(eds), 2020; Dodigovic and Agustín-Llach, 2020). Furthermore, despite some limitations in sampling procedures and being concerned with only receptive knowledge of vocabulary, the current study is an example of the efforts made by language teachers to connect findings from corpus-based studies of language to the vocabulary learning needs of EFL learners. Such undertakings might be more effective if mobile assisted learning becomes more integrated into instructional curricula and teaching methodologies. Further research might consider the impacts of using DFs on developing other aspects of vocabulary knowledge such as productive use of learned items or their collocations.

## Data Availability Statement

The original contributions presented in the study are included in the article/supplementary material; further inquiries can be directed to the corresponding author.

## Ethics Statement

Ethical review and approval was not required for the study on human participants in accordance with the local legislation and institutional requirements. The patients/participants provided their written informed consent to participate in this study.

## Author Contributions

All authors contributed to the design and implementation of the research, to the analysis of the results, and to the writing of the manuscript.

## Conflict of Interest

The authors declare that the research was conducted in the absence of any commercial or financial relationships that could be construed as a potential conflict of interest.

## Publisher’s Note

All claims expressed in this article are solely those of the authors and do not necessarily represent those of their affiliated organizations, or those of the publisher, the editors and the reviewers. Any product that may be evaluated in this article, or claim that may be made by its manufacturer, is not guaranteed or endorsed by the publisher.
